# Development and Comparison of Two Multiresidue Methods for the Determination of 17 *Aspergillus* and *Fusarium* Mycotoxins in Cereals Using HPLC-ESI-TQ-MS/MS

**DOI:** 10.3389/fmicb.2019.00361

**Published:** 2019-03-04

**Authors:** Valentina Scarpino, Amedeo Reyneri, Massimo Blandino

**Affiliations:** Department of Agricultural, Forest and Food Sciences, University of Turin, Turin, Italy

**Keywords:** deoxynivalenol, deoxynivalenol-3-glucoside, fumonisins, aflatoxins, enniatins, moniliformin, zearalenone

## Abstract

Cereals can be contaminated by several mycotoxins, whose co-presence may represent an undervalued risk for humans and animals. Maize and wheat are the most contaminated cereals and in temperate areas could be affected in field conditions by several *Fusarium* and *Aspergillus* infections. To date, only B-fumonisins (FBs), aflatoxins (AFs), zearalenone (ZEA), deoxynivalenol (DON) and T-2 and HT-2 toxins have been regulated in cereals in European Union. The other fungal metabolites, are commonly referred to as “emerging” and “masked” mycotoxins, and more information on their occurrence in combination with the regulated mycotoxins, are needed to design combined toxicological and exposure assessments.

This research intends to develop and compare two multiresidue HPLC-ESI-TQ-MS/MS methods for the simultaneous determination of the main regulated, emerging and masked mycotoxins in maize and wheat, among which: FB_1_, FB_2_, DON, ZEA, AFB_1_, AFB_2_, AFG_1_, AFG_2_, moniliformin (MON), deoxynivalenol-3-glucoside (DON-3-G), 3-acetyldeoxynivalenol (3-ADON), 15-acetyldeoxynivalenol (15-ADON), nivalenol (NIV), enniatins A, A_1_, B, B_1_ (ENNA, ENNA_1_, ENNB, ENNB_1_). The extraction was performed for both methods using a mixture of CH_3_CN/H_2_O/CH_3_COOH (79/20/1, v/v/v), while the dilution/purification was carried out through two different procedures: (1) by the “dilute-and-shoot” technique diluting 1:2 the filtered extract with CH_3_CN/H_2_O/CH_3_COOH (20/79/1, v/v/v) to reduce the matrix effect; (2) using the Oasis^®^ PRiME HLB clean-up columns. The analysis was carried out using CH_3_OH and H_2_O both acidified with 0.1% of CH_3_COOH as eluents. The injection volume was 20 μL and the flow rate 200 μL min^-1^. The analysis of two reference material (maize and wheat), was performed to evaluate the trueness and precision of the two methods by matrix-matched calibration curves. For all the regulated mycotoxins analyzed by both methods, the range of recovery percentage established by the Regulation (EC) No. 401/2006 was respected, except for ZEA by using the Oasis^®^ PRiME HLB clean-up columns. Nevertheless, the results suggest that the Oasis^®^ PRiME HLB clean-up columns, could be a valid alternative to the dilute-and-shoot method, although an additional cost for the clean-up has to be considered. In conclusion, both two analytical methods considerably reduce the analytical time and costs and therefore result to be promising and applicable for high-throughput routine multi-mycotoxins analysis by the use of a TQ.

## Introduction

Mycotoxins are secondary metabolites that are produced by microfungi which are capable of causing disease and death in humans and other animals (Bennett and Klich, 2003).

According to a risk assessment overview provided by the European Food Safety Authority (EFSA) (EFSA CONTAM Panel) related to the main contaminants in food and feed, mycotoxins represented 15% of the overall risk for human and animal health, for the period between 2003 and 2012 (EFSA, 2012).

Mycotoxins are produced by several fungal species and they can affect agricultural commodities, causing worldwide yield and economic losses (Steyn, 1995), due to their negative impact on the safety and quality of these commodities.

Five mycotoxin classes are considered to be of great economic and toxicological importance for grain in several areas throughout the world: aflatoxins (AFBs) and ochratoxin (OTA), produced by the *Aspergillus* and *Penicillium* genera, deoxynivalenol (DON), zearalenone (ZEA), and B-fumonisins (FBs), which are mainly produced by *Fusarium* species (Atkins and Norman, 1998).

Among the agricultural commodities affected by mycotoxins, cereals are the most contaminated (Placinta et al., 1999) and in particular maize and wheat, which, in temperate areas, could mainly be affected, in field conditions, by fungal disease caused by several *Fusarium* species (Logrieco et al., 2002). These *Fusarium* species are the main cause of FB and type-B trichothecene DON production and accumulation, in maize and in wheat, in temperate areas.

The incidence and the concentration of different mycotoxins is clearly variable over the years and from area to area through the world. In fact, mycotoxin contamination depends on the co-existence of host susceptibility and environmental conditions that are favorable to fungal infection, growth and toxinogenesis (Munkvold, 2003). Unfortunately, since the same plant tissue may be colonized by various mycotoxigenic species, it is possible that several mycotoxins could co-occur in the same food or feed matrix, with consequent possible additive or synergic toxicological effects due to their co-presence (Sanhueza and Degrossi, 2004).

The toxicological effects of the mycotoxins that are regulated by the European Commission [EC] (2007, 2010) are well known, but only few data are available in literature about toxicological studies of their combined toxicity. Lee and Ryu (2017) summarized the most relevant early studies that reported additive or synergistic effects due to the co-occurrence of mycotoxins and their interactive toxicity.

Any other mycotoxins and fungal metabolites which till now have not received detailed scientific attention, are commonly referred to as “novel” or “emerging” mycotoxins such as moniliformin (MON), enniatins (ENNs), nivalenol (NIV), etc. (Streit et al., 2013). Furthermore, in addition to the emerging mycotoxins it is also important to remember the “masked” mycotoxins which are the fraction of biologically modified mycotoxins that were conjugated by plants resulting from plant defense reactions after fungal infection which can have higher toxicity (i.e., 15-acetyldeoxynivalenol (15-ADON)) or be released, hydrolyzed, biotransformed and absorbed (i.e., deoxynivalenol-3-glucoside (DON-3-G)) in the gastrointestinal tract, primarily as the parent compound and should be considered an additional contributing factor of the total dietary exposure to the native form (i.e., DON) (Berthiller et al., 2013). The EFSA is currently working on gathering data for establishing a scientific opinion on the risks to public health related to the presence of emerging and masked mycotoxins in feed and food (EFSA, 2014, 2017, 2018).

Little is known about the toxicological effects of these compounds, some of which could be potentially toxic to humans and livestock. The main risks are in particular related to their additive or synergistic effects, if present together with regulated mycotoxins, for which their toxicological effects have mainly been defined regarding their individual presence.

In order to avoid an underestimated increased level of risk for the end-consumer, there is an urgent need to develop accurate, precise and sensitive multi-residue analytical methods but which are also fast and easily applicable in routine analysis of both regulated and emerging mycotoxins in order to acquire data on their co-occurrence.

So far, due to the different chemical properties of mycotoxins, their routine analysis has usually been performed through the determination of single compound one by one or, of certain classes of mycotoxins, through multi-determination by means of HPLC coupled with non-confirmatory UV or fluorescence detectors (Krska and Molinelli, 2007). For these reasons, liquid chromatography-tandem mass spectrometry (LC-MS/MS) has become a prominent tool for multiresidue analysis over the last few years, as pointed out in the updates published by Berthiller et al. (2015, 2016, 2017). Since LC-MS/MS methods suffer from matrix effects, the most common difficulties in developing multiresidue methods concern finding a compromise between reducing the matrix effects as much as possible, using different clean-up procedures, and increasing the number of the mycotoxins surveyed over a wide range of polarities. Most of the multiresidue LC-MS/MS methods reported in literature have adopted an intensive clean-up with sequential solid-phase extraction (SPE) (Biselli and Hummert, 2005; Klötzel et al., 2006), which limits the number of surveyed mycotoxins and is highly time-consuming, or other clean-up procedures such as accelerated solvent extraction (ASE) (Royer et al., 2004), the QuEChERS-like method (Desmarchelier et al., 2010), or the more versatile dilute-and-shoot type methods (Sulyok et al., 2006; Spanjer et al., 2008; Beltrán et al., 2009; Malachova et al., 2014), that offer the opportunity of extending the number of surveyed mycotoxins by reducing or even avoiding the sample clean-up, but could compromise the quantification (LOQ value). In addition, the applicability of all the previously described clean-up procedures, except for the dilute-and-shoot approaches, to the simultaneous analysis of ENNs and MON has yet to be proven, and is probably not feasible (Sulyok et al., 2006).

In this scenario, the current research is aimed at investigating the applicability of the developed multiresidue analytical methods to a high-throughput routine analysis for the screening and confirmation of the main EU-regulated and most relevant emerging mycotoxins to which EFSA has paid attention through the use of a TQ. For this purpose, two fast, reliable and repeatable multiresidue HPLC-ESI-MS/MS methods were developed and compared for the quantitative determination of: AFB_1_, AFB_2_, AFG_1_, AFG_2_, 3-acetyldeoxynivalenol (3-ADON), 15-ADON, DON, DON-3-G, enniatins A, A_1_, B, B_1_ (ENNA, ENNA_1_, ENNB, ENNB_1_), FB_1_, FB_2_, MON, NIV, and ZEA in maize and wheat.

## Materials and Methods

### Chemicals and Reagents

Methanol (CH_3_OH), acetonitrile (CH_3_CN), and water (H_2_O) were LC gradient grade or LC-MS grade, depending on their use during the extraction or the analytical phases, and were purchased from VWR (Milan, Italy). Glacial acetic acid (CH_3_COOH) was obtained from Sigma-Aldrich (St. Louis, MO, United States).

**FIGURE 1:**
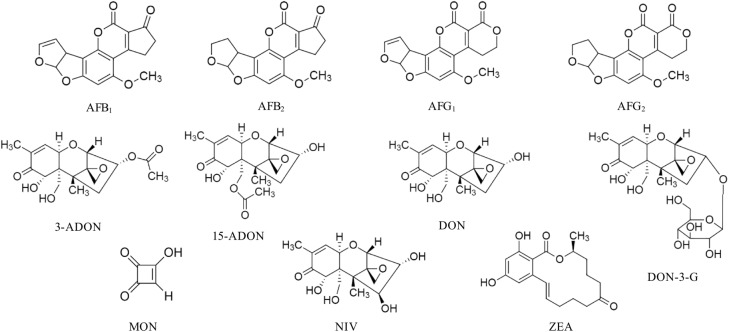
Chemical structure of aflatoxin B_1_ (AFB_1_), aflatoxin B_2_ (AFB_2_), aflatoxin G_1_ (G_1_), aflatoxin G_2_ (AFG_2_), 3-acetyldeoxynivalenol (3-ADON), 15-acetyldeoxynivalenol (15-ADON), deoxynivalenol (DON), deoxynivalenol-3-glucoside (DON-3-G), moniliformin (MON), nivalenol (NIV), and zearalenone (ZEA).

Mycotoxin standards were purchased from different sources and were dissolved in CH_3_CN, if not stated otherwise. Stock solutions of AFB_1_, AFB_2_, AFG_1_, AFG_2_, 3-ADON, 15-ADON, DON, DON-3-G, FB_1_, FB_2_, in CH_3_CN/H_2_O 50/50, v/v; MON, in CH_3_CN/H_2_O 90/10, v/v; NIV and ZEA were purchased from Romer Labs Diagnostic GmbH (Tulln, Austria). ENNA, ENNA_1_, ENNB, ENNB_1_ were instead obtained from Sigma-Aldrich (St. Louis, MO, United States). The chemical structure of the studied mycotoxins is reported in Figure 1, 2. Two composite standard working solutions were prepared by dissolving appropriate volumes of each analyte in a diluting phase mixture, CH_3_CN/H_2_O 50/50, v/v as follows: the first working solution contained AFB_1_, AFB_2_, AFG_1_, AFG_2_, DON, DON-3-G, FB_1_, FB_2_, MON, and ZEA and the second one contained 3-ADON, 15-ADON, ENNA, ENNA_1_, ENNB, ENNB_1_, and NIV. These two working solutions were then mixed in appropriate volumes and dissolved in CH_3_CN/H_2_O/CH_3_COOH 49.5/49.5/1 v/v/v in order to prepare the working solutions for the calibration. All the solutions were stored at -20°C in amber glass vials and were brought to room temperature before use.

**FIGURE 2:**
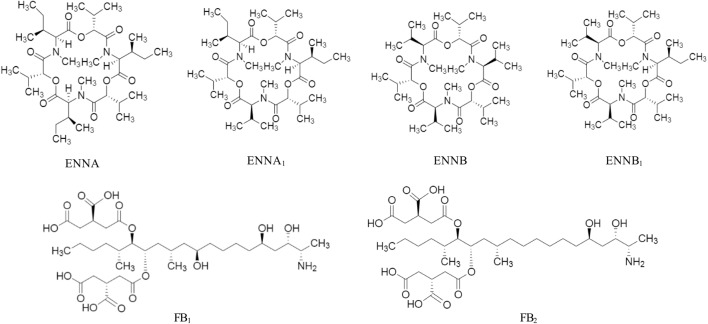
Chemical structure of enniatin A (ENNA), enniatin A_1_ (ENNA_1_), enniatin B (ENNB), enniatin B_1_ (ENNB_1_), fumonisin B_1_ (FB_1_), and fumonisin B_2_ (FB_2_).

### Samples

The reference materials of maize (AFB_1_, AFB_2_, AFG_1_, AFG_2_, DON, FB_1_, FB_2_, ZEA) and wheat (DON), containing certified concentrations of mycotoxins, as well as blank wheat and maize samples were purchased from Trilogy^®^ Analytical Laboratory (Washington, MO, United States). All the samples were already available as fine powder and therefore did not require any further grinding. Blank maize and wheat samples (2.5 g) were spiked, for validation purposes, by adding appropriate amounts of the two combined working solutions, and they were kept overnight at room temperature to allow the integration and equilibration of the analytes in the respective matrices checking before extraction that all the amount of the solvent evaporated.

### Sample Preparation

Two multi-residue methods were tested and compared, and each sample preparation is described hereafter.

#### Dilute-and-Shoot Method

The dilute-and-shoot method was performed by applying the procedure described by Sulyok et al. (2006) with slight modifications. Five grams of maize or wheat flour weighted into a 50 mL centrifuge tube was extracted by mechanical shaking at 300 rpm for 90 min (shaker mod. RS-LS 20, Phoenix Instrument, Garbsen, Germany) with 20 mL of CH_3_CN/H_2_O/CH_3_COOH (79/20/1, v/v/v). The extract was filtered through Whatman^®^ grade 1 filters (Brentford, United Kingdom) and subjected to dilution with the same volume of diluting solution (CH_3_CN/H_2_O/CH_3_COOH 20/79/1, v/v/v) in order to reduce SSE due to matrix effects. The diluted extract was vortexed and filtered through 15 mm diameter, 0.2 μm regenerated cellulose (RC) syringe filters (Phenex-RC, Phenomenex, Torrance, CA, United States). After appropriate mixing, 20 μL of the diluted filtered extract was injected into the liquid chromatography, which was coupled with a tandem mass spectrometry (LC-MS/MS) system without any further pre-treatment.

#### Oasis^®^ PRiME HLB Method

A 5 g cereal sample (maize or wheat) was weighted into a 50 mL centrifuge tube and 20 mL of the extracting solution (CH_3_CN/H_2_O/CH_3_COOH 79/20/1, v/v/v) was added. The extraction was performed for 90 min at 300 rpm using a mechanical shaker (shaker mod. RS-LS 20, Phoenix Instrument, Garbsen, Germany). The extract was filtered through Whatman^®^ grade 1 filters (Brentford, United Kingdom) and passed through a clean-up Oasis^®^ PRiME HLB cartridge (Waters Corporation, Milford, MA, United States) to remove the fats and phospholipids. An Oasis^®^ PRiME HLB cartridge (3 cc, 150 mg) was mounted onto a pre-cleaned vacuum manifold, set at minimum vacuum conditions (approximately 2 psi). No cartridge conditioning was required or performed, and a 0.4 mL aliquot of the filtered extract was passed through the cartridge and discarded. A 1 mL aliquot of the filtered extract was then passed through the cartridge and collected. After appropriate mixing, 20 μL of the diluted filtered extract was injected into the LC-MS/MS system.

### LC-MS/MS Analysis

LC-MS/MS analysis was carried out on a Varian 310 triple quadrupole (TQ) mass spectrometer (Varian, Italy), equipped with an electrospray ionization (ESI) source, a 212 LC pump, a ProStar 410 AutoSampler and dedicated software. Liquid chromatography (LC) separation was performed on a Gemini-NX C_18_ 100 × 2.0 mm i.d., 3 μm particle size, 110 Å equipped with a C_18_ 4 × 2 mm security guard cartridge column (Phenomenex, Torrance, CA, United States). The mobile phase consisted of two eluents: H_2_O (eluent A) and CH_3_OH (eluent B), both of which were acidified with 0.1% v/v CH_3_COOH delivered at 200 μL min^-1^. In order to quantify all the analytes with positive and negative polarity, two separate chromatographic runs per sample were carried out. The run for the negative ionization mode acquisition took 15 min and consisted of the following gradient: after an initial time of 2 min at 90% A, the proportion of B was increased linearly to 100% in 2 min, and this was followed by a hold time of 2 min at 100% B and 4 min column re-equilibration at 90% A; finally, the initial condition at 90% A was kept for 5 min before the subsequent chromatographic run. The positive ionization mode acquisition run instead took 13 min and consisted of the following gradient: the elution was started with 90% A and the proportion of B was increased linearly over 2 min to 100%; this condition was kept for 2 min and B was then decreased linearly to the initial condition of 10% over 5 min and kept at this level for 4 min in order to re-equilibrate the column.

Mass spectrometric analysis (ESI-MS/MS) was performed, in selected reaction monitoring (SRM) mode, alternating two transition reactions for each compound in both negative and in positive ionization modes in two separate chromatographic runs per sample with the following settings: the nebulizing gas was N_2_ (20 psi); the drying gas was air (250°C in negative ionization mode and 300°C in positive ionization mode, 25 psi); the needle voltage was -4000 V and +5000 V, the shield voltage was -600 V and +600 V and the detector voltage was -1950 V and +1950 V, for negative and positive polarity, respectively; the collision gas was Ar (2.00 mTorr).

MS tuning and the optimization of the analyte-dependent MS/MS parameter was performed by means of direct syringe-infusion of a separate standard solution of each analyte into the TQ using a 11 Plus syringe pump (Harvard Apparatus, Holliston, MA, United States) at a flow rate of 10 μL min^-1^.

### Calibration Curves

In order to evaluate the performance of the method and validate both of the tested methods, a set of three different calibration curves was prepared in triplicate as follows:

(1) External calibration in neat solvent: for external calibration, a multi-analyte stock solution was prepared freshly by mixing the two combined working solutions at appropriate amounts with CH_3_CN/H_2_O/CH_3_COOH 49.5/49.5/1 v/v/v. This solution was further diluted with CH_3_CN/H_2_O/CH_3_COOH 49.5/49.5/1 v/v/v to obtain the same concentration levels as the following two matrix-matched calibration curves;
(2) Matrix-matched calibration curve in a blank extract: this type of matrix-matched calibration curve was built for each cereal matrix (maize and wheat) at six different concentration levels for DON, ENNA, ENNA_1_, ENNB, ENNB_1_, FB_1_, FB_2_, MON and ZEA, at five different concentration levels for 3-ADON, 15-ADON, DON-3-G and NIV, and at four different concentration levels for AFB_1_, AFB_2_, AFG_1_ and AFG_2_ by spiking appropriate volumes of mycotoxin working solutions, after extraction, into blank sample extracts to obtain the concentration levels reported in the ‘Results’ section;
(3) Matrix-matched calibration curve in sample extracts: this type of matrix-matched calibration curve was built for each cereal matrix (maize and wheat) at six different concentration levels for DON, ENNA, ENNA_1_, ENNB, ENNB_1_, FB_1_, FB_2_, MON and ZEA, at five different concentration levels for 3-ADON, 15-ADON, DON-3-G and NIV, and at four different concentration levels for AFB_1_, AFB_2_, AFG_1_ and AFG_2_ by spiking appropriate volumes of mycotoxin working solutions, before extraction, into blank samples to obtain the concentration levels reported in the ‘Results’ section.

### Performance of the Methods

The performance of both of the analytical methods was evaluated, for each mycotoxin, using the previously described set of three calibration curves, and the following parameters were assessed: the linearity range, the limit of detection (LOD); the limit of quantification (LOQ), the matrix effect, considering signal suppression/enhancement SSE (%) and the recovery of the extraction *R*_E_ (%), through the evaluation of the apparent recovery *R*_A_ (%). The analyses were conducted in triplicate for all the parameters in order to evaluate the repeatability (Relative Standard Deviation of Repeatability, RSD_r_). Furthermore, the analysis of two certified matrices (Trilogy^®^ Reference Material, Trilogy^®^ Analytical Laboratory, Washington, MO, United States), one of wheat and one of maize, respectively, with a certified concentration of DON in the wheat and of AFBs, DON, ZEA and FBs in the maize, was performed in order to evaluate the trueness and precision of the two methods through a quantification with matrix-matched calibration curves.

The LOD and LOQ were defined for each mycotoxin as the concentrations which yielded measure peaks with a signal-to-noise ratio (S/N) of 3 and 10, respectively, and were calculated by injecting (*n* = 5) neat solvent standard solutions at different concentration levels. The LOQ were then verified and validated using the calculated value as the lowest validated level in the spiking experiment.

The *R*_A_, SSE due to the matrix effects and *R*_E_ were calculated from the previously described six points calibration curves as follows (Matuszewski et al., 2003):

$$R_{A}(\%)=100\times {slope}_{\text{spiked sample}}/{slope}_{\text{neat solvent}}$$

$$SSE(\%)=100\times {slope}_{\text{spiked extract}}/{slope}_{\text{neat solvent}}$$

$$R_{E}(\%)=100\times R_{A}/SSE$$

The formula of recovery as just reported was in agreement with the IUPAC nomenclature and was chosen to distinguish between incomplete extraction of the analytes and the effects/losses that arose from ion suppression/enhancement due to the matrix.

## Results

### Optimization of the Extraction and Sample Preparation Methods

As far as the dilute-and-shoot method is concerned, the main deviation from the original method (Sulyok et al., 2006) essentially concerned the replacement of the filtration step with Whatman^®^ grade 1 filters instead of the centrifugation step after the extraction, and addition of a further filtration step, after the dilution step, with a 15 mm diameter, 0.2 μm regenerated cellulose (RC) syringe filters.

The Oasis^®^ PRiME HLB method was applied, without the additional QuEChERS extraction and dispersive SPE (dSPE) steps.

Preliminary tests were also performed using MycoSpin^TM^ 400 clean-up columns, but these tests were not continued because the MON signal in the spiked samples disappeared after the clean-up step, in spite of satisfactory results having been recorded for all the other analyzed mycotoxins.

The extracting solution was acidified with 1% of CH_3_COOH, for both methods, to obtain a satisfactory recovery of FB_1_ and FB_2_.

### HPLC-ESI-TQ-MS/MS Optimization

First, the MS and MS/MS parameters (selection of the most abundant SRM transitions and the adduct ions used as precursor ions, declustering potentials and collision energy) were optimized for all the mycotoxins in both positive and negative ESI mode. Table 1 summarizes the optimized SRM transition parameters. Tuning experiments were used to choose the best ionization mode and to select the most intense adduct ions as both precursor and product ions. Most of the analytes showed a relatively high attitude to ionize in both modes, and reasonably high signal intensities were recorded for both the precursor ions and product ions. However, some mycotoxins, including MON and NIV, gave no or very weak signals in positive ion mode, whereas it was no possible to apply the negative mode for the ENNs. Accordingly, the ESI+ and the ESI- modes were set in two separate chromatographic runs per sample to guarantee the optimal MS/MS conditions for all the analytes. A better ionization yield was recorded using ESI+ and it was therefore selected for AFB_1_, AFB_2_, AFG_1_, AFG_2_, 15-ADON, ENNA, ENNA_1_, ENNB, ENNB_1_, FB_1_ and FB_2_, whereas the use of ESI- was favored for 3-ADON, DON, DON-3-G, MON, NIV and ZEA. The formation of particular adducts was taken into consideration for both the precursor and product ions during the optimization step for each analyte and these adduct ions were selected if the intensity of the signal associated with each SRM improved. All the mycotoxins analyzed in ESI+ formed [M+H]^+^, while on the other hand both [M-H]^-^ and [M+CH_3_COOH]^-^ were formed in ESI-, depending on the considered mycotoxin. MON and ZEA formed [M-H]^-^, whereas 3-ADON, DON, DON-3-G and NIV formed the acetate adduct with a higher signal intensity than the loss of a hydrogen ion. Collision-induced dissociation (CID) experiments were then conducted to select at least two SRMs per analyte, with the exception of MON, which showed only one product ion. The more intense of these two fragmentation pathways was used for quantification purposes (quantifier product ion), whereas the less intense one was selected for identification purposes (qualifier product ion). In addition, the analyte peaks from both of the product ions in the extracted ion chromatograms fully overlapped, and the ion ratio variations measured in the sample extracts were within ±30% of the requested performance criteria listed in SANTE/11813/2017, 2017. The optimized LC-ESI-MS/MS was also compliant with the minimum requirement of including at least three identification points (retention time, molecular mass, one characteristic product ion) in the method to confirm the substances listed in group B of Annex I of Directive 96/23/EC (European Commission [EC], 2002).

**Table 1:** Optimized ESI-MS and ESI-MS/MS parameters and monitored transition reactions for the analyzed mycotoxins.

Mycotoxin	Retention time (min)	Precursor ion (*m/z*)	Adduct ion	Declustering potential (V)	Product ions*^a^* (*m/z*)	Collision energy (V)
AFB_1_	4.12	313.1	[M+H]^+^	80	241.1/213.3	37/45
AFB_2_	4.05	315.2	[M+H]^+^	80	259.0/286.9	26/20
AFG_1_	4.00	329.1	[M+H]^+^	80	243.4/215.4	26/33
AFG_2_	3.98	331.1	[M+H]^+^	80	245.1/313.1	30/22
3-ADON	6.02	397.3	[M+CH_3_COO]^-^	-65	336.9/306.9	-8/-13
15-ADON	3.91	339.1	[M+H]^+^	35	137.1/339.1	12/7
DON	5.67	355.2	[M+CH_3_COO]^-^	-35	265.0/294.9	-12/-10
DON-3-G	5.55	517.3	[M+CH_3_COO]^-^	-60	427.0/457.1	-18/-10
ENNA	5.86	682.7	[M+H]^+^	60	210.2/228.3	26/27
ENNA_1_	5.77	668.7	[M+H]^+^	60	210.1/228.2	29/28
ENNB	5.43	640.7	[M+H]^+^	60	196.2/214.2	28/27
ENNB_1_	5.60	654.7	[M+H]^+^	60	196.2/214.3/210.5	28/27/26
FB_1_	3.79	722.5	[M+H]^+^	80	334.3/352.3	39/35
FB_2_	4.03	706.5	[M+H]^+^	80	336.4/318.4	37/39
MON	11.02	96.9	[M-H]^-^	-40	41.1	-13
NIV	5.40	371.2	[M+CH_3_COO]^-^	-40	280.8/310.9	-13/-9
ZEA	6.84	317.1	[M-H]^-^	-80	174.7/130.4	-24/-29


**^a^Numerical value are given in the order quantifier/qualifier (/second qualifier).**

**FIGURE 3:**
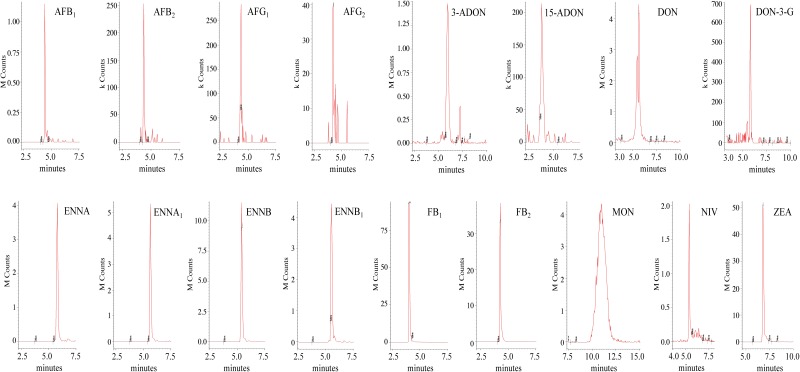
LC-ESI-MS/MS chromatograms of mycotoxins from maize extract spiked at 8 μg kg^-1^ for AFB_1_, 2 μg kg^-1^ for AFG_1_ and AFB_2_, 1 μg kg^-1^ for AFG_2_, 100 μg kg^-1^ for 3-ADON, FB_2_ and MON, 40 μg kg^-1^ for 15-ADON and NIV, 168 μg kg^-1^ for DON, 50 μg kg^-1^ for DON-3-G, 32.4 μg kg^-1^ for ENNA, 36 μg kg^-1^ for ENNA_1_, 28 μg kg^-1^ for ENNB, 30.4 μg kg^-1^ ENNB_1_, 500 μg kg^-1^ FB_1_, 120 μg kg^-1^ ZEA.

**FIGURE 4:**
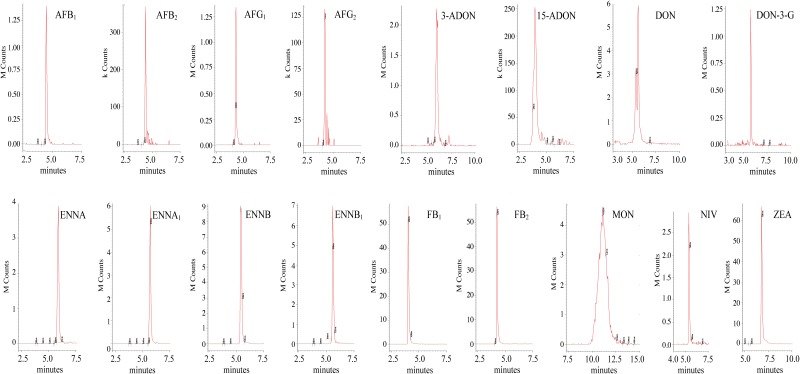
LC-ESI-MS/MS chromatograms of mycotoxins from wheat extract spiked at 8 μg kg^-1^ for AFB_1_, 2 μg kg^-1^ for AFG_1_ and AFB_2_, 1 μg kg^-1^ for AFG_2_, 100 μg kg^-1^ for 3-ADON, FB_2_ and MON, 40 μg kg^-1^ for 15-ADON and NIV, 168 μg kg^-1^ for DON, 50 μg kg^-1^ for DON-3-G, 32.4 μg kg^-1^ for ENNA, 36 μg kg^-1^ for ENNA_1_, 28 μg kg^-1^ for ENNB, 30.4 μg kg^-1^ ENNB_1_, 500 μg kg^-1^ FB_1_, 120 μg kg^-1^ ZEA.

As far as the chromatographic separation is concerned, the chosen mobile phases were CH_3_OH and H_2_O, both of which were acidified with 0.1% of CH_3_COOH. CH_3_OH was preferred to CH_3_CN for sensitivity reasons. The addition of CH_3_COOH to both mobile phases increased the overall sensitivity and led to a better peak shape of the acidic compounds, i.e., FB_1_ and FB_2,_ due to the presence of four carboxylic groups in their molecular structure. Moreover, it was decided not to add any buffer (ammonium acetate or ammonium formate) to the mobile phases because a decrease in the signal was recorded when it was added. A Gemini-NX reversed-phase C_18_ column was chosen as the stationary phase, because it exhibited reasonable peak shapes of all the analytes, despite their chemical diversity. The LC-ESI-MS/MS SRM chromatograms of the spiked maize and wheat samples are reported in Figure 3, 4, respectively.

### Performance of the Methods

Linearity was tested for both of the compared methods by evaluating the determination coefficients (*R*^2^), and for all of the prepared calibration curves (in a neat solvent, in a spiked blank extract and in a spiked blank sample) in both the maize and wheat matrices. The concentration ranges in which the linearity was tested included for each studied mycotoxin the LOQ value as the lower limit of the range and an estimate, based on data reported in literature, of the highest values of concentration commonly found in natural contaminated cereal samples in temperate areas, as the upper limit of the range. An *R*^2^ > 0.99 was recorded for all the targeted mycotoxins, in all of the tested matrices and for both of the methods.

The results pertaining to the linearity range, the LOD, the LOQ, the apparent recovery *R*_A_ (%), the matrix effects through the evaluation of the SSE (%) and the recovery of the extraction *R*_E_ (%) are reported for all the analyzed mycotoxins in Tables 2, 3 for the maize and wheat matrices, respectively.

**Table 2:** Method performance parameter in maize (*n* = 3) for both of the compared methods.

Mycotoxin	Clean-up method	Linearity range (μg kg^-1^)	LOD (μg kg^-1^)	LOQ (μg kg^-1^)	Spike levels (μg kg^-1^)	*R*_A_ ± RSD_r_ (%)	SSE ± RSD_r_ (%)	*R*_E_ ± RSD_r_ (%)
AFB_1_	Dilute-and-shoot	1.6–80	0.5	1.6	1.6, 4, 8, 40	48 ± 6	46 ± 6	105 ± 10
	Oasis® PRiME HLB	1.6–80	0.5	1.6	1.6, 4, 8, 40	44 ± 14	39 ± 8	114 ± 10
AFB_2_	Dilute-and-shoot	1–20	0.3	1	1, 2, 10	41 ± 11	43 ± 7	96 ± 17
	Oasis® PRiME HLB	1–20	0.3	1	1, 2, 10	40 ± 1	43 ± 13	94 ± 13
AFG_1_	Dilute-and-shoot	1.6–80	0.5	1.6	1.6, 4, 8, 40	37 ± 4	36 ± 26	106 ± 20
	Oasis® PRiME HLB	1.6–80	0.5	1.6	1.6, 4, 8, 40	27 ± 19	26 ± 13	105 ± 24
AFG_2_	Dilute-and-shoot	1–20	0.3	1	1, 2, 10	51 ± 4	53 ± 1	96 ± 3
	Oasis® PRiME HLB	1–20	0.3	1	1, 2, 10	32 ± 11	30 ± 10	108 ± 22
3-ADON	Dilute-and-shoot	10–2000	3	10	10, 40, 100, 200, 1000	46 ± 17	76 ± 15	62 ± 1
	Oasis® PRiME HLB	10–2000	3	10	10, 40, 100, 200, 1000	36 ± 4	51 ± 9	71 ± 4
15-ADON	Dilute-and-shoot	10–2000	3	10	10, 40, 100, 200, 1000	45 ± 11	45 ± 3	101 ± 14
	Oasis® PRiME HLB	10–2000	3	10	10, 40, 100, 200, 1000	52 ± 2	47 ± 19	112 ± 15
DON	Dilute-and-shoot	4.2–8400	1.2	4.2	4.2, 42, 168, 420, 840, 4200	49 ± 9	59 ± 16	83 ± 7
	Oasis® PRiME HLB	4.2–8400	1.2	4.2	4.2, 42, 168, 420, 840, 4200	44 ± 22	48 ± 5	90 ± 19
DON-3-G	Dilute-and-shoot	10–1000	3	10	10, 20, 50, 100, 500	39 ± 10	59 ± 1	65 ± 8
	Oasis® PRiME HLB	10–1000	3	10	10, 20, 50, 100, 500	29 ± 6	38 ± 7	76 ± 3
ENNA	Dilute-and-shoot	1.6–1620	0.5	1.6	1.6, 8.1, 32.4, 81, 162, 810	62 ± 12	87 ± 26	73 ± 14
	Oasis® PRiME HLB	1.6–1620	0.5	1.6	1.6, 8.1, 32.4, 81, 162, 810	72 ± 12	109 ± 4	65 ± 9
ENNA_1_	Dilute-and-shoot	1.8–1800	0.6	1.8	1.8, 9, 36, 90, 180, 900	86 ± 20	92 ± 14	93 ± 6
	Oasis® PRiME HLB	1.8–1800	0.6	1.8	1.8, 9, 36, 90, 180, 900	104 ± 14	114 ± 6	92 ± 20
ENNB	Dilute-and-shoot	1.4–1400	0.4	1.4	1.4, 7, 28, 70, 140, 700	99 ± 13	101 ± 3	98 ± 10
	Oasis® PRiME HLB	1.4–1400	0.4	1.4	1.4, 7, 28, 70, 140, 700	85 ± 19	101 ± 16	84 ± 4
ENNB_1_	Dilute-and-shoot	1.5–1520	0.5	1.5	1.5, 7.6, 30.4, 76, 152, 760	87 ± 14	91 ± 5	96 ± 9
	Oasis® PRiME HLB	1.5–1520	0.5	1.5	1.5, 7.6, 30.4, 76, 152, 760	92 ± 14	110 ± 2	85 ± 15
FB_1_	Dilute-and-shoot	5–10000	1.5	5	5, 50, 200, 500, 1000, 5000	64 ± 1	84 ± 10	76 ± 8
	Oasis® PRiME HLB	5–10000	1.5	5	5, 50, 200, 500, 1000, 5000	41 ± 6	61 ± 1	67 ± 6
FB_2_	Dilute-and-shoot	2–2000	0.6	2	2, 10, 40, 100, 200, 1000	69 ± 3	64 ± 2	109 ± 5
	Oasis® PRiME HLB	2–2000	0.6	2	2, 10, 40, 100, 200, 1000	45 ± 11	66 ± 2	67 ± 9
MON	Dilute-and-shoot	1–2000	0.3	1	1, 10, 40, 100, 200, 1000	67 ± 9	89 ± 11	76 ± 15
	Oasis® PRiME HLB	1–2000	0.3	1	1, 10, 40, 100, 200, 1000	63 ± 12	83 ± 19	76 ± 10
NIV	Dilute-and-shoot	10–2000	3	10	10, 40, 100, 200, 1000	35 ± 19	42 ± 6	82 ± 15
	Oasis® PRiME HLB	10–2000	3	10	10, 40, 100, 200, 1000	19 ± 5	30 ± 1	63 ± 6
ZEA	Dilute-and-shoot	1.2–2400	0.4	1.2	1.2, 12, 48, 120, 240, 1200	54 ± 11	85 ± 17	65 ± 11
	Oasis® PRiME HLB	1.2–2400	0.4	1.2	1.2, 12, 48, 120, 240, 1200	40 ± 28	98 ± 26	41 ± 8


**Table 3:** Method performance parameter in wheat (*n* = 3) for both of the compared methods.

Mycotoxin	Clean-up method	Linearity range (μg kg^-1^)	LOD (μg kg^-1^)	LOQ (μg kg^-1^)	Spike levels (μg kg^-1^)	R_A_ ± RSD_r_ (%)	SSE ± RSD_r_ (%)	*R*_E_ ± RSD_r_ (%)
AFB_1_	Dilute-and-shoot	1.6–80	0.5	1.6	1.6, 4, 8, 40	87 ± 28	83 ± 16	105 ± 20
	Oasis® PRiME HLB	1.6–80	0.5	1.6	1.6, 4, 8, 40	82 ± 22	69 ± 26	121 ± 12
AFB_2_	Dilute-and-shoot	1–20	0.3	1	1, 2, 10	109 ± 29	101 ± 15	106 ± 16
	Oasis® PRiME HLB	1–20	0.3	1	1, 2, 10	73 ± 9	60 ± 15	125 ± 22
AFG_1_	Dilute-and-shoot	1.6–80	0.5	1.6	1.6, 4, 8, 40	90 ± 8	78 ± 24	117 ± 17
	Oasis® PRiME HLB	1.6–80	0.5	1.6	1.6, 4, 8, 40	74 ± 8	70 ± 13	107 ± 11
AFG_2_	Dilute-and-shoot	1–20	0.3	1	1, 2, 10	100 ± 4	78 ± 4	128 ± 1
	Oasis® PRiME HLB	1–20	0.3	1	1, 2, 10	73 ± 5	55 ± 2	134 ± 2
3-ADON	Dilute-and-shoot	10–2000	3	10	10, 40, 100, 200, 1000	66 ± 4	76 ± 12	87 ± 9
	Oasis® PRiME HLB	10–2000	3	10	10, 40, 100, 200, 1000	39 ± 9	57 ± 3	69 ± 13
15-ADON	Dilute-and-shoot	10–2000	3	10	10, 40, 100, 200, 1000	70 ± 7	59 ± 4	119 ± 4
	Oasis® PRiME HLB	10–2000	3	10	10, 40, 100, 200, 1000	57 ± 16	55 ± 37	110 ± 22
DON	Dilute-and-shoot	4.2–8400	1.2	4.2	4.2, 42, 168, 420, 840, 4200	66 ± 6	73 ± 17	92 ± 16
	Oasis® PRiME HLB	4.2–8400	1.2	4.2	4.2, 42, 168, 420, 840, 4200	44 ± 7	58 ± 4	76 ± 5
DON-3-G	Dilute-and-shoot	10–1000	3	10	10, 20, 50, 100, 500	54 ± 14	68 ± 9	80 ± 16
	Oasis® PRiME HLB	10–1000	3	10	10, 20, 50, 100, 500	36 ± 8	43 ± 25	82 ± 28
ENNA	Dilute-and-shoot	1.6–1620	0.5	1.6	1.6, 8.1, 32.4, 81, 162, 810	69 ± 25	76 ± 5	91 ± 20
	Oasis® PRiME HLB	1.6–1620	0.5	1.6	1.6, 8.1, 32.4, 81, 162, 810	64 ± 4	98 ± 1	66 ± 4
ENNA_1_	Dilute-and-shoot	1.8–1800	0.6	1.8	1.8, 9, 36, 90, 180, 900	94 ± 20	93 ± 4	101 ± 23
	Oasis® PRiME HLB	1.8–1800	0.6	1.8	1.8, 9, 36, 90, 180, 900	91 ± 8	104 ± 12	89 ± 20
ENNB	Dilute-and-shoot	1.4–1400	0.4	1.4	1.4, 7, 28, 70, 140, 700	105 ± 12	101 ± 5	104 ± 17
	Oasis® PRiME HLB	1.4–1400	0.4	1.4	1.4, 7, 28, 70, 140, 700	86 ± 11	85 ± 11	102 ± 1
ENNB_1_	Dilute-and-shoot	1.5–1520	0.5	1.5	1.5, 7.6, 30.4, 76, 152, 760	100 ± 10	95 ± 2	106 ± 12
	Oasis® PRiME HLB	1.5–1520	0.5	1.5	1.5, 7.6, 30.4, 76, 152, 760	92 ± 13	102 ± 2	90 ± 15
FB_1_	Dilute-and-shoot	5–10000	1.5	5	5, 50, 200, 500, 1000, 5000	27 ± 6	66 ± 7	41 ± 11
	Oasis® PRiME HLB	5–10000	1.5	5	5, 50, 200, 500, 1000, 5000	30 ± 33	67 ± 14	44 ± 26
FB_2_	Dilute-and-shoot	2–2000	0.6	2	2, 10, 40, 100, 200, 1000	45 ± 16	72 ± 17	63 ± 8
	Oasis® PRiME HLB	2–2000	0.6	2	2, 10, 40, 100, 200, 1000	55 ± 18	73 ± 14	75 ± 16
MON	Dilute-and-shoot	1–2000	0.3	1	1, 10, 40, 100, 200, 1000	62 ± 7	98 ± 17	65 ± 13
	Oasis® PRiME HLB	1–2000	0.3	1	1, 10, 40, 100, 200, 1000	61 ± 19	95 ± 24	65 ± 7
NIV	Dilute-and-shoot	10–2000	3	10	10, 40, 100, 200, 1000	41 ± 5	60 ± 1	68 ± 5
	Oasis® PRiME HLB	10–2000	3	10	10, 40, 100, 200, 1000	22 ± 2	35 ± 1	61 ± 3
ZEA	Dilute-and-shoot	1.2–2400	0.4	1.2	1.2, 12, 48, 120, 240, 1200	68 ± 5	81 ± 12	85 ± 17
	Oasis® PRiME HLB	1.2–2400	0.4	1.2	1.2, 12, 48, 120, 240, 1200	37 ± 30	88 ± 18	42 ± 12


The extent of SSE was quite different for the different mycotoxins in the two matrices. High values of signal suppression were recorded in the maize (Table 2) for the AFs, 15-ADON and NIV for both of the sample preparation methods and for 3-ADON, DON, DON-3-G, but only with the Oasis® PRiME HLB method. On the other hand, heavy signal suppression effects were observed in wheat for DON-3-G and NIV, but only with the Oasis® PRiME HLB method.

The *R*_E_ for all the analyzed mycotoxins in maize was in the 62–106% range when the dilute-and-shoot method was applied, whereas when the Oasis® PRiME HLB method was used, all the mycotoxins showed an *R*_E_ range of 63–114%, with the exception of ZEA, which recorded an *R*_E_ value of 41%. The *R*_E_ ranges recorded in the wheat matrix as a result of the application of the dilute-and-shoot method and the Oasis® PRiME HLB method were 63–128% and 61–125%, respectively, for all of the detected mycotoxins, with the exception of FB_1_ (41% and 44% for the two methods, respectively) and again for ZEA, for which an *R*_E_ value of 42% was recorded when the Oasis® PRiME HLB method was applied.

The RSD_r_ obtained for all the targeted mycotoxins in both matrices and for both methods was always lower than 30%, and even lower than 20% for almost all of the combined tested models.

The results of the analysis of two certified matrices (Trilogy® Reference Material), one of wheat and one of maize, respectively, with a certified concentration of DON in the wheat and of AFBs, DON, ZEA, and FBs in the maize are reported in Table 4. In maize reference material in addition to the certified mycotoxins other mycotoxins have been detected by both methods, among which: 3-ADON, 15-ADON, DON-3-G, and MON. Similarly, in wheat reference material in addition to DON both methods also detected 3-ADON, 15-ADON, DON-3-G, ENNB, ENNB_1_, MON, and ZEA. Almost all the measured concentrations for the certified mycotoxins in maize and wheat reference materials obtained through both analytical methods showed a high degree of agreement with the certified concentration recording on average a deviation from the true value <20%.

**Table 4:** Comparison between the certified (CC) and measured concentrations (MC) (*n* = 3) of the certified maize and wheat reference material (Trilogy® Reference Material, Trilogy® Analytical Laboratory, Washington, MO, United States).

Mycotoxin	Maize			Wheat		
		
	CC ± SD (μg kg^-1^)	MC ± SD Dilute-and-shoot (μg kg^-1^)	MC ± SD Oasis® PRiME HLB (μg kg^-1^)	CC ± SD (μg kg^-1^)	MC ± SD Dilute-and-shoot (μg kg^-1^)	MC ± SD Oasis® PRiME HLB (μg kg^-1^)
AFB_1_	11.2 ± 1.7	11.7 ± 0.1	11.8 ± 0.3	–	<LOD	<LOD
AFB_2_	1.0 ± 0.1	1.2 ± 0.2	1.1 ± 0.2	–	<LOD	<LOD
AFG_1_	<LOD*^a^*	<LOD	<LOD	–	<LOD	<LOD
AFG_2_	<LOD*^a^*	<LOD	<LOD	–	<LOD	<LOD
3-ADON	-	30 ± 2	31 ± 1	–	75 ± 13	69 ± 4
15-ADON	-	88 ± 2	97 ± 14	–	160 ± 3	170 ± 3
DON	1400 ± 100	1423 ± 203	1498 ± 41	2900 ± 200	2959 ± 12	2902 ± 97
DON-3-G	-	274 ± 40	233 ± 44	-	405 ± 11	406 ± 31
ENNA	-	<LOD	<LOD	-	<LOD	<LOD
ENNA_1_	-	<LOD	<LOD	-	<LOD	<LOD
ENNB	-	<LOD	<LOD	-	16 ± 2	16 ± 1
ENNB_1_	-	<LOD	<LOD	-	7 ± 2	9 ± 1
FB_1_	2000 ± 214	2194 ± 69	2195 ± 88	-	<LOD	<LOD
FB_2_	600 ± 64	724 ± 43	735 ± 19	-	<LOD	<LOD
MON	–	1860 ± 66	1830 ± 108	-	40 ± 12	24 ± 7
NIV	–	<LOD	<LOD	-	<LOD	<LOD
ZEA	174 ± 16	182 ± 17	169 ± 13	-	26 ± 10	24 ± 13


**^a^LOD = 0.5 μg kg^-*1*^.**

## Discussion

Multiresidue LC-MS/MS methods have become a fundamental tool to investigate the overall contamination as a result of the co-occurrence of mycotoxins in the same sample, and to provide important data to design new, combined toxicological and exposure assessments.

Over the last few years, several multi-mycotoxin LC-MS/MS methods have been proposed in literature (Sulyok et al., 2006; Ren et al., 2007; Frenich et al., 2009; Desmarchelier et al., 2010; Malachova et al., 2014; De Santis et al., 2017) for different targeted mycotoxins and uses.

In the present experiment, it was decided to compare two modified sample preparation procedures that were considered to limit the preparation steps of the sample as much as possible with the aim of being faster, and therefore applicable for routine analysis, and of guaranteeing a powerful and reliable tool to simultaneously analyze multiple mycotoxins in the same feed and food matrix. The mycotoxins that were chosen for inclusion in the developed methods were both the regulated mycotoxins that are commonly found in cereals and the main emerging and masked mycotoxins to which EFSA has paid attention and requested occurrence data, that is, MON, DON-3-G, 3-ADON, 15-ADON, ENNs (EFSA, 2014, 2017, 2018). To date, the inclusion of the previous mycotoxins in a multi-mycotoxins method together with the regulated mycotoxins was only be proposed in few researches in which the analysis was carried out by more advanced and performing mass spectrometers (Sulyok et al., 2006; Malachova et al., 2014).

To the best of the authors’ knowledge, this is the first time that Oasis® PRiME HLB columns have been applied for the clean-up in a multiresidue analysis of the mycotoxins considered in the present survey. Moreover, their use was always associated with other steps, such as QuEChERS extraction and dSPE steps, which allow a greater purification of the matrix from proteins, starch and polar sugars, but which also lengthen the analytical time and increase the propagation of errors. As a result of the elimination of these two steps, the procedure applied in the present survey has become more comparable with the dilute-and-shoot method, while ensuring shorter analytical times and satisfactory results in terms of precision, accuracy and reliability. Although Desmarchelier et al. (2010) reported that a defatting step with *n*-exane, followed by a two-step sequential reconstitution in CH_3_OH/H_2_O, could be a valid alternative to Oasis® HLB columns, Oasis® PRiME HLB columns remain one of the best choices to move further toward green analytical chemistry, or at least to limit the use of chemical reagents during the analytical steps, if a fast clean up step in the sample purification method is desired.

As far as chromatographic separation is concerned, the HPLC mobile phases commonly used for mycotoxin analysis are composed of CH_3_OH and H_2_O acidified with acetic or formic acid and/or buffered with acetate or a formate buffer. CH_3_OH is usually favored over CH_3_CN, for sensitivity reasons (Berthiller et al., 2005). These acids are generally added in order to increase the ionization efficiency in the ESI source and to obtain a better peak shape for acidic compounds, whereas a buffer could aid the chromatographic separation and the sensitivity of some compounds that show more attitude to form adduct ions (Sulyok et al., 2006). No buffer additions have been considered in the developed methods because they were shown to cause ESI ion suppression of some of the detected mycotoxins. Furthermore, although other studies (Delmulle et al., 2006; Sulyok et al., 2006) reported the use of 5 mM ammonium acetate in the mobile phases, their results showed high noises in chromatograms, similarly to those obtained in the present survey. It was instead considered essential to acidify both of the mobile phases in order to increase the overall sensitivity and to obtain a better peak shape of FB_1_ and FB_2._ All mycotoxins were detected, through a fast determination, in 28 min applying two separate chromatographic runs, one in negative and one in positive ionization mode acquisition, took 15 min and 13 min, respectively. However, it was not possible to avoid the co-elution of some mycotoxins and, as previously reported (Delmulle et al., 2006; Sulyok et al., 2006), the co-elution was accepted because the related compounds show different transitions.

The recovery percentage range established by Regulation (European Commission [EC], 2006). No. 401/2006 was respected for all the legislated mycotoxins determined through the use of both of the sample preparation methods and for both cereal matrices, except for ZEA when the Oasis® PRiME HLB method was used. The low *R*_E_ values of FB_1_ in the wheat are of limited relevance for the evaluation of the applicability of the methods, because fumonisins are the main maize mycotoxins and they were never found on the wheat, if not in traces. The recovery values were in agreement with previous report (Delmulle et al., 2006; Sulyok et al., 2006; Spanjer et al., 2008). Moreover, the application of both of the developed methods to the analysis of the maize and wheat reference materials, certified for their mass concentrations of DON in wheat and of AFBs, DON, ZEA, and FBs in maize, showed that the experimentally determined concentrations were in agreement with the certified values for both methods, and even revealed and quantified the presence of several other mycotoxins within these matrices, thus confirming a real and concrete occurrence of some of these mycotoxins.

The quantification was conducted by means of matrix-matched calibration curves, because they were considered the best alternative to the too expensive isotopically labeled internal standard (which was not available for all the analyzed mycotoxins) to compensate for both losses during extraction and matrix effects generated during the ionization of the analytes. This quantification procedure, although tested successfully, still requires the availability of matrices that are free of any of the surveyed mycotoxins, or with a contamination as low as possible.

The LOQ values assessed for AFB_1_, AFB_2_, AFG_1_, and AFG_2_ were high compared to other methods (Sulyok et al., 2006; Malachova et al., 2014; De Santis et al., 2017), and this was above all due to the high dilution factor of the two methods (eight for the dilute-and-shoot method and four for the Oasis® PRiME HLB method), that is not compatible with not-last-generation TQ, due to its lower instrumental sensitivity than other mass spectrometers.

The two developed analytical methods offer different advantages and suffer from different disadvantages. The main advantages of the dilute-and-shoot method are the low costs and the extremely reduced sample preparation times, while the main disadvantage is related to the instrumental detection limit that can be achieved with a not-last-generation TQ. On the other hand, the main advantage of the clean-up method that uses the Oasis® PRiME HLB columns is a higher purification level of the sample, which protects the instrument more from becoming dirty, while the main disadvantage is that, in order to obtain the same results and analytical performance for the different determined mycotoxins, an additional cost is foreseen for the clean-up columns.

Nevertheless, in conclusion the results suggest that the two analytical methods and both of the sample preparation techniques considerably reduce the analytical time and costs and therefore result to be both promising and applicable for high-throughput routine analysis through the use of a TQ, in order to collect data on the co-occurrence of both regulated and the most relevant among emerging and masked mycotoxins in cereals.

## Data Availability

All datasets generated for this study are included in the manuscript and/or the supplementary files.

## Author Contributions

VS and MB conceived and designed the experiments. VS performed the experiments and run laboratory analysis. VS analyzed the data. VS contributed reagents, materials, and analysis tools. VS wrote the main manuscript text. All authors reviewed the manuscript.

## Conflict of Interest Statement

The authors declare that the research was conducted in the absence of any commercial or financial relationships that could be construed as a potential conflict of interest.

## Abbreviations

3-ADON: 3-acetyldeoxynivalenol
15-ADON: 15-acetyldeoxynivalenol
AFB_1_: aflatoxin B_1_, AFB_2_, aflatoxin B_2_
AFG_1_: aflatoxin G_1_
AFG_2_: aflatoxin G_2_
DON: deoxynivalenol
DON-3-G: deoxynivalenol-3-glucoside
EC: European Commission
EFSA: European Food Safety Authority
ENNA: enniatin A
ENNA_1_: enniatin A_1_
ENNB: enniatin B
ENNB_1_: enniatin B_1_
ESI: electrospray ionization
FB_1_: fumonisin B_1_
FB_2_: fumonisin B_2_
HPLC: high performance liquid chromatography
LC-MS/MS: liquid chromatography coupled to tandem mass spectrometry detection
LOD: limit of detection
LOQ: limit of quantification
MON: moniliformin
NIV: nivalenol
*R*_A_: apparent recovery
*R*_E_: recovery of extraction
RSD_r_: relative standard deviation of repeatability
S/N: signal-to-noise ratio
SSE: signal suppression/enhancement
*t*_R_: retention time
TQ: triple quadrupole
ZEA: zearalenone
